# Case Report: The first reported case of pulmonary alveolar proteinosis with myasthenia gravis in a 27-year-old patient

**DOI:** 10.12688/f1000research.127299.1

**Published:** 2022-12-06

**Authors:** Lenda Ben Hmida, Islam Mejri, Maroua Kacem, Mariem Msalmani, Hana Blibech, Houda Snène, Aida Ayadi, Jamel Zaouali, Zied Moatemri

**Affiliations:** 1 Pneumology Department, Military Hospital of Tunis, Montfleury, Tunis, 1008, Tunisia; 2 Neurology Department, Military Hospital of Tunis, Montfleury, Tunis, 1008, Tunisia; 3 Pneumology Department, Mongi Slim Hospital, La Marsa, Tunis, 2070, Tunisia; 4 Pathology Department, Abderrahmen Mami Hospital, Ariana, 2080, Tunisia

**Keywords:** Pulmonary alveolar proteinosis, Myasthenia gravis, GM-CSF antibodies, Bronchoalveolar lavage, Whole lung lavage, Rituximab

## Abstract

**Background:** Pulmonary alveolar proteinosis is a very rare diffuse lung disease characterized by the accumulation of amorphous and periodic acid Schiff-positive lipoproteinaceous material in the alveolar spaces due to impaired surfactant clearance by alveolar macrophages. Three main types were identified: Autoimmune, secondary and congenital. Pulmonary alveolar proteinosis has been previously reported to be associated with several systemic auto-immune diseases. Accordingly, we present the first case report of pulmonary alveolar proteinosis associated with myasthenia gravis.

**Case:** A 27-year-old female patient, ex-smoker, developed a dyspnea on exertion in 2020. The chest X-ray detected diffuse symmetric alveolar opacities. Pulmonary infection was ruled out, particularly COVID-19 infection. The chest scan revealed the “crazy paving” pattern. The bronchoalveolar lavage showed a rosy liquid with granular acellular eosinophilic material Periodic acid-Schiff positive. According to the lung biopsy results, she was diagnosed with pulmonary alveolar proteinosis. The granulocyte macrophage colony-stimulating factor autoantibodies were negative. Nine months later, she was diagnosed with bulbar seronegative myasthenia gravis, confirmed with the electroneuromyography with repetitive nerve stimulation showing significant amplitude decrement of the trapezius and spinal muscles. She was treated with pyridostigmine, oral corticosteroids and azathioprine. Given the worsening respiratory condition of the patient, a bilateral whole lung lavage was performed with a partial resolution of symptoms. Thus, this previously unreported association was treated successfully with rituximab, including improvement of dyspnea, diplopia and muscle fatigability at six months of follow-up.

**Conclusions:** This case emphasizes on the possible association of auto-immune disease to PAP, which could worsen the disease course, as the specific treatment does not exist yet. Hence, further studies are needed to establish clear-cut guidelines for PAP management, particularly when associated to auto-immune diseases.

## Introduction

Pulmonary alveolar proteinosis (PAP), also known as pulmonary alveolar phospholipoproteinosis, is a very rare chronic diffuse lung disease. ^1^ It is characterized by the accumulation of amorphous and Periodic acid-Schiff (PAS)-positive lipoproteinaceous material in the alveolar spaces due to impaired surfactant clearance by alveolar macrophages. ^2^ However, the underlying lung structure is preserved. ^2^ The lipoproteinaceous material is principally composed of abnormal surfactant phospholipids and apoproteins. ^1^ The accumulated substances filling the alveoli results in damaging gas exchange. ^1^ The classic symptoms are dyspnea and hypoxemia, ultimately leading to respiratory failure and death. ^1^ There are three main types of PAP: Autoimmune (previously named primary or idiopathic), secondary and congenital. ^1^ PAP has been previously reported to be associated with several systemic auto-immune diseases. ^2^ Accordingly, we present the first case report of PAP associated with myasthenia gravis (MG).

## Case report

A 27-year-old, North African female patient, had a medical history of allergic rhinosinusitis and asthma. She is working as a web editor, mainly as a work from home employee. Her tobacco consumption amounted to one pack per day over two years. She had no particular exposure to toxics. On July 2020, she had an acute onset of respiratory symptoms, which consisted of productive cough, dyspnea on exertion and fever. She was, empirically, treated as a respiratory infection with antibiotics. One month later, she remained with a dyspnea occurring with effort. The physical examination and the laboratory findings were unremarkable. The chest X-ray detected diffuse symmetric alveolar opacities. Pulmonary infection was ruled out, particularly COVID-19 infection.

**Figure 1.**
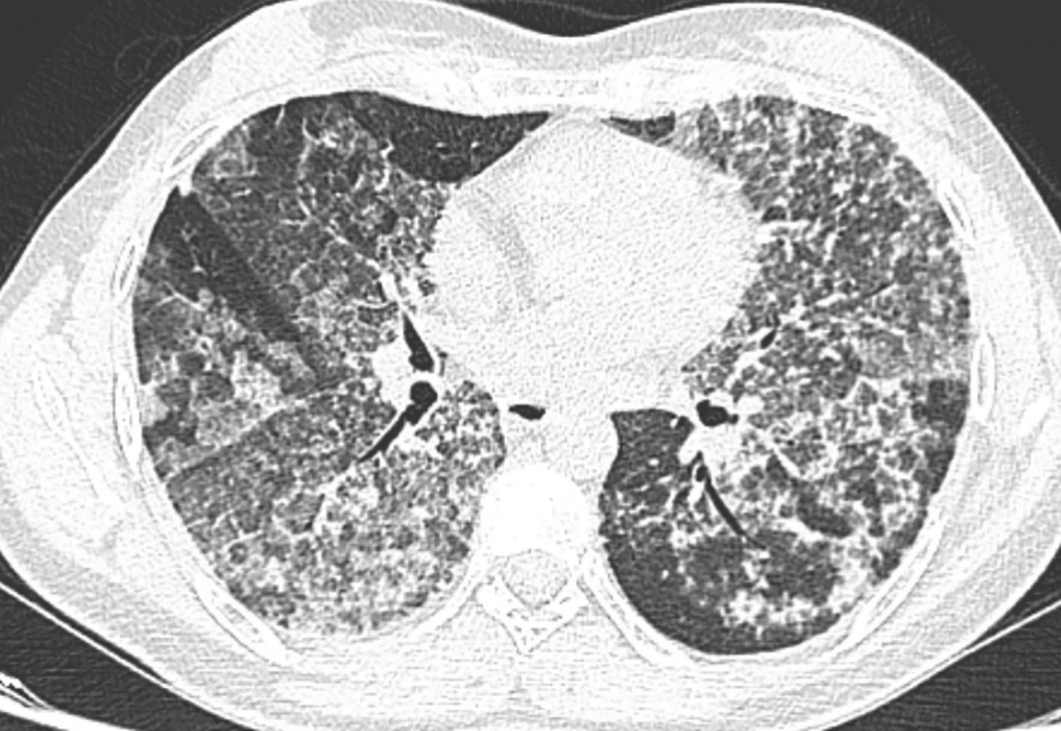
Bilateral diffuse ground-glass opacities with interlobular and intralobular septal thickening, forming the “crazy paving” pattern.

**Figure 2.**
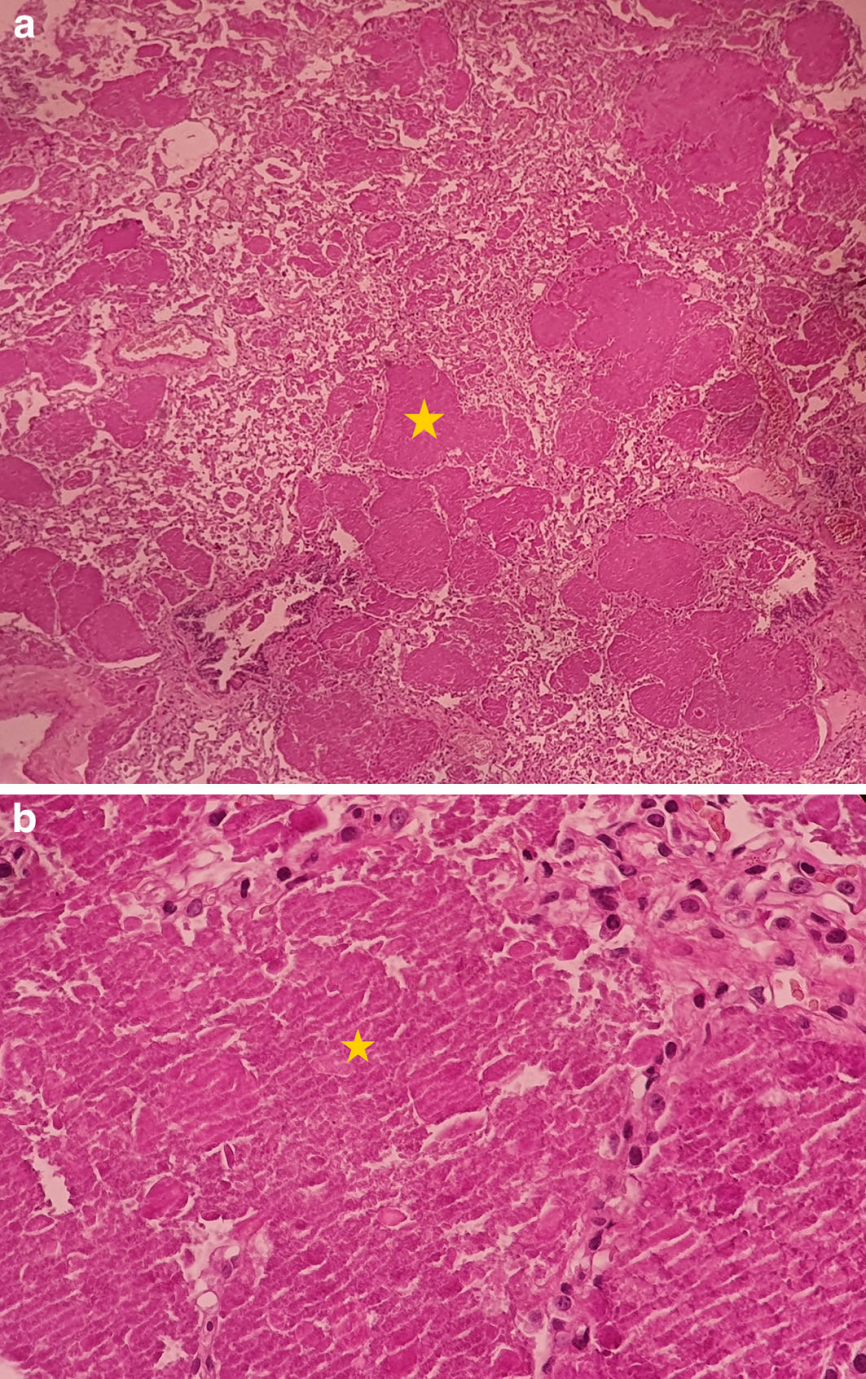
Pulmonary alveoli filled with eosinophilic, acellular, granular and PAS-positive material. The yellow * indicates lipoproteinaceous material containing foamy macrophages. (a): low magnification (×4); (b): high magnification (×40). PAS, Periodic acid-Schiff.

The chest scan revealed bilateral ground-glass peri-broncho-vascular opacities with interlobular and intralobular septal thickening, defining the “crazy paving” pattern ( Figure 1). The bronchoalveolar lavage showed a rosy liquid with granular acellular eosinophilic material PAS positive. The lung biopsy confirmed the diagnosis of PAP showing alveoli filled with eosinophilic, acellular, granular and PAS-positive material containing foamy macrophages and some cholesterol crystals ( Figure 2). At the time of diagnosis, the granulocyte macrophage colony-stimulating factor (GM-CSF) autoantibodies were not available. Lung function tests were normal, apart from a declined diffusing capacity of lung for carbon monoxide (<68% of the predicted value). Laboratory results, including quantitative immunoglobulins, proteins electrophoresis and autoantibody screening were normal, apart from an elevated c-antineutrophil cytoplasmic antibodies and anti-Mi-2 antibodies without clinical signification.

Nine months after the onset, the patient presented with asthenia, muscle fatigability and right diplopia with worsening of symptoms later in the day. An electroneuromyography with repetitive nerve stimulation showed a significant amplitude decrement of the trapezius and spinal muscles. The patient, thus was diagnosed with bulbar MG. The acetylcholine receptor antibodies were negative. The patient was treated with pyridostigmine (60 mg pill, three times a day, for life), oral corticosteroids (prednisone 50 mg/day, once a day, for 5 weeks followed by progressive degression to 15 mg/day) and azathioprine (50 mg pill, twice a day, for 8 months). At that time, GM-CSF autoantibodies were negative.

**Figure 3.**
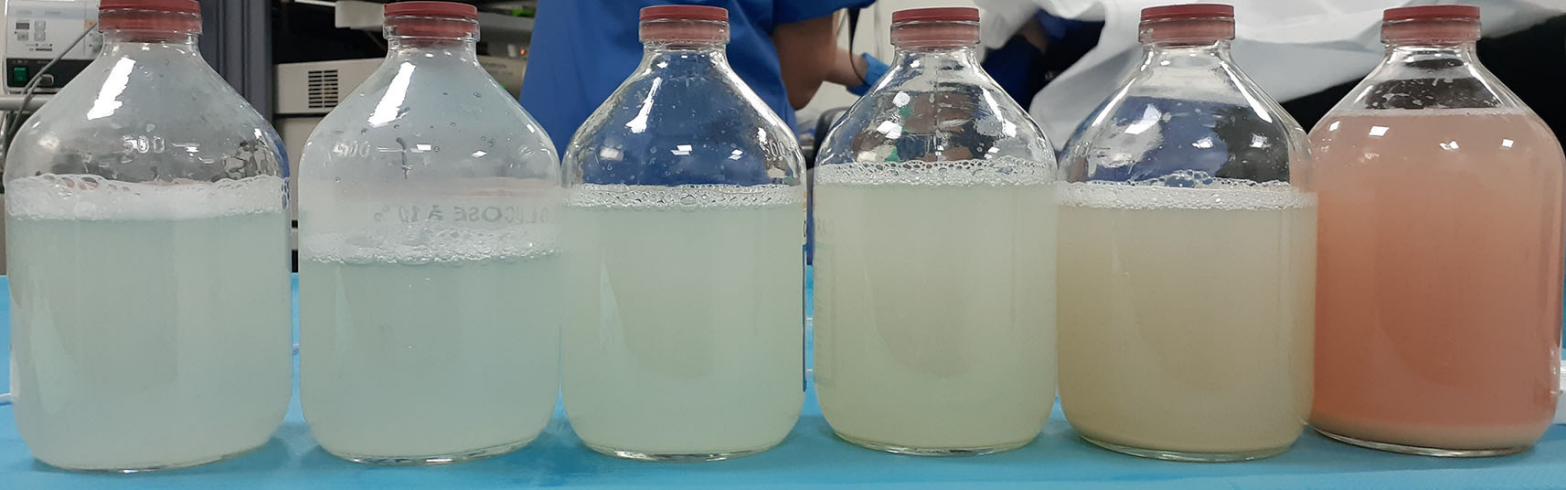
Alveolar liquid derived from whole lung lavage of the left lung (10 L) gradually turning clearer.

**Figure 4.**
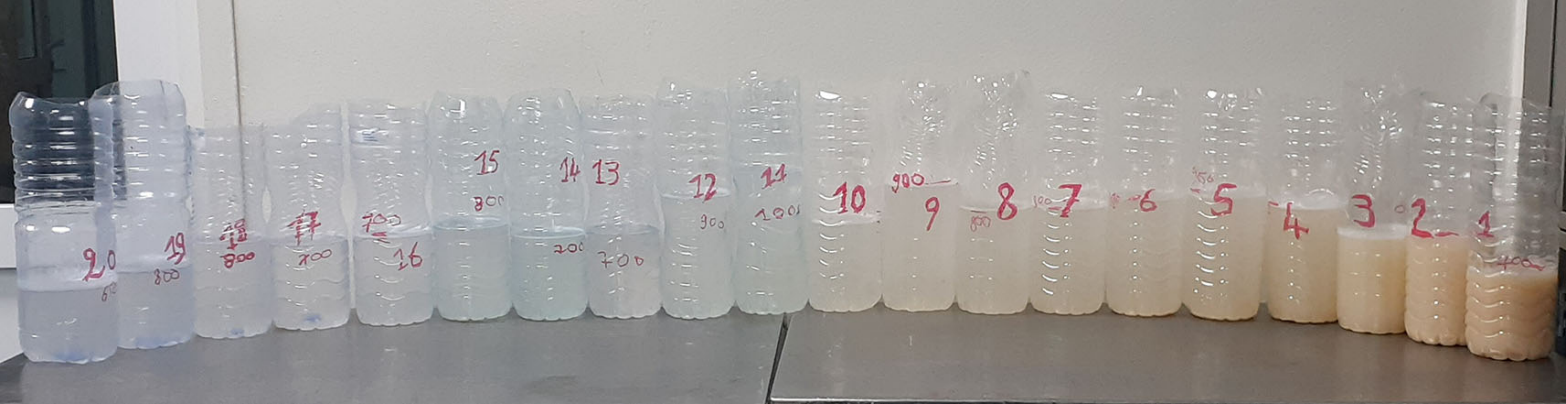
Alveolar liquid derived from whole lung lavage of the right lung (20 L) gradually turning clearer.

During the follow-up period, the patient’s respiratory condition worsened. She presented with oxygen desaturation at the level of 70% after a 6-min walk of 100 meters. Lung function tests degraded further, with a severe alteration of alveolocapillary diffusion (diffusing capacity of lung for carbon monoxide at 33% of the predicted value). After excluding the main differential diagnosis, the patient underwent a whole lung lavage (WLL). The left lung was washed with 10 liters of saline ( Figure 3). Six weeks later, the right lung was washed with 20 liters ( Figure 4). There was a partial resolution of symptoms. Following a multidisciplinary discussion, the patient was treated with rituximab (two intravenous injections of 1,000 mg, 14 days apart), after 6 weeks of azathioprine washout. An improvement of dyspnea, diplopia and muscle fatigability was noted at six months of follow-up.

## Discussion

PAP is an ultra-rare alveolar filling process with an estimated prevalence of 6.87 per million in the general population. ^1^ PAP was first described by Rosen in 1958 and back to 2009, only 500 cases were reported in the literature. ^1^^,^^3^ PAP occurs mainly in men with a sex ratio of two and a typical age of 40 to 50 years old in adults, which is contrasting with our case. ^2^ About 50 to 80% of patients with PAP have a smoking history, as it was reported in our patient. ^2^ The symptoms are non-specific, subacute and mild, resulting often in delaying the diagnosis by months, even by years. ^3^ The positive diagnosis of PAP is suggested by the chest scan findings with the classically known “crazy paving” pattern. ^3^ Bronchoalveolar lavage and the transbronchial biopsy with the characteristic features, establish the diagnosis of PAP. ^3^ In the present case, PAP was confirmed by the pathological typical results of a lung surgical biopsy. Based on the pathogenic mechanism, PAP can be grouped into three types. ^1^ Firstly, primary PAP as the most frequent form found in 95% of patients, is an autoimmune disease caused by elevated levels of the GM-CSF autoantibodies. ^1^ In our patient, the GM-CSF antibodies were negative. However, their assessment was conducted under corticosteroids and azathioprine. The autoimmune hypothesis, though, was not definitely ruled out, especially when an associated auto-immune disease appeared during the course of PAP. Secondly, secondary PAP, occurring in 5% of patients, results from alveolar macrophage dysfunction caused by immune dysregulation, hematopoietic disorders, environmental exposure and pharmaceutical agents. ^1^ In our case there was no other associated underlying illness or exposure, which eliminated a secondary PAP. Thirdly, congenital PAP occurs due to genetic variations, usually observed in children, which is not our case. ^1^ In the current case, the onset of symptoms began after a respiratory infection that was very likely the initial trigger causing probably abnormal response in surfactant uptake. ^4^ In the literature, there have been several cases of PAP associated with systemic auto-immune diseases; such as hemolytic anemia, polymyalgia rheumatica, ulcerative colitis, granulomatous polyangiitis, systemic lupus erythematosus and dermatomyositis. ^4^^,^^5^ In this regard, our case is noteworthy as it demonstrates the first case in the literature of PAP and MG association.

Our patient had a confirmed seronegative MG and was undergoing treatment with corticosteroids and an immunosuppressant. Whether the MG or its treatment has any bearing on the progression of PAP is questionable. Indeed, the worsening condition of the patient after MG treatment, suggested that the immunosuppressant therapy could be the exacerbating factor of PAP. This hypothesis is plausible, as it was reported in the literature. In fact, three cases of patients with collagen disease developing autoimmune PAP during the immunosuppressant therapy were reported. ^5^ Nagasawa *et al.*, outlined the development of autoimmune PAP, in a patient previously diagnosed with systemic lupus erythematosus, under glucocorticoid therapy and its worsening under immunosuppressive therapy. ^6^ As for the treatment of PAP, WLL is the standard of care. ^1^ However no randomized controlled trials have been reported on WLL due to the extreme rarity of PAP. ^1^ New therapeutic strategies for PAP have emerged, including GM-CSF, rituximab and plasmapheresis. ^1^ Rituximab is an anti-CD20, already used in several autoimmune diseases with a proven efficiency. ^7^ A clinical trial with rituximab, conducted by Kavuru *et al.*, included 10 patients with PAP, had shown promise in seven out of nine patients. ^7^ In our case, a bilateral WLL was performed with a partial resolution of symptoms. Considering the exercise intolerance persistence after WLL and taking into account the negative level of GM-CSF antibodies; our patient was treated with rituximab, which resulted in promising outcomes. This result supports the auto-immune PAP hypothesis.

In conclusion, this case emphasizes the possible association between auto-immune diseases and PAP, which could worsen the disease course, as the specific treatment does not exist yet. Hence, further observational studies and randomized controlled trials are needed to establish clear-cut guidelines for PAP management, particularly when associated with auto-immune diseases.

## Consent

Written informed consent for publication of their clinical details and clinical images was obtained from the patient.

## Data Availability

All data underlying the results are available as part of the article and no additional source data are required.
