# Supplementary material for: Case Report: The first reported case of pulmonary alveolar proteinosis with myasthenia gravis in a 27-year-old patient
Source: F1000Res. 2023 Aug 21;11:1439. Originally published 2022 Dec 6. [Version 2] doi: 10.12688/f1000research.127299.2 (PMC11109554; doi:10.12688/f1000research.127299.2)
Supplement: Peer review history

# Reviewer report: Reviewer response for version 2

**DOI:** 10.5256/f1000research.154600.r210778

**Published:** 2024-05-14

**Authors:** Iván Oterino-Moreira

**Affiliations:** 1 Pharmacy Department, Alcorcon Foundation University Hospital, Alcorcon, Spain

The authors present an interesting case of association between two probably autoimmune diseases in the same patient: primary pulmonary alveolar proteinosis (aPAP) and myasthenia gravis (MG).

 This is a fact widely studied in recent years, in which patients with immune dysfunction develop more than one disease of autoimmune origin affecting various organs/tissues. The greatest exponent is autoimmune polyglandular syndrome, in which several unrelated hormone-producing glands are autoimmunely affected (Addison's disease, autoimmune thyroid disease and/or type 1 diabetes) and even primary hypogonadism, myasthenia gravis or celiac disease[ https://rarediseases.info.nih.gov/diseases/7611/autoimmune-polyendocrinopathy-type-2].^^

 aPAP is the most common presentation of PAP, and in 90% of cases it presents with GM-CSF neutralizing antibodies that produce dysfunction of the alveolar macrophage, limiting the clearance of pulmonary surfactant, causing deficiencies in gas exchange ^1^.In this case, the anti GM-CSF antibodies are negative, so the diagnosis of aPAP is made by ruling out, as it cannot be related to secondary or tertiary PAP (according to the authors). Sometimes, an infectious disease can trigger a subsequent autoimmune disease.

 The authors present the case following the international “CARE” consensus at the request of reviewer 1. However, these guidelines recommend a detailed and objective description of the patient's situation before and after the therapeutic intervention. The authors talk about improvement or worsening of clinical parameters without showing the objective value (for example, they talk about “improvement in dyspnea” but not about how many points they improve on the mRCO dyspnea scale).

 The authors should construct a table showing the clinical and/or functional evolution parameters of the disease from the diagnosis of aPAP, worsening after diagnosis of MG and performing whole lung lavage (WLL), after WLL (treatment of choice), new worsening and after of the administration of rituxumab (although these are early results, they are useful for the scientific community given the status of experimental therapy)

 Examples of these parameters are respiratory function tests (FVC, FFEV1, RV, DLCO, KCO), six-minute walk test (theoretical distance, HR minute 6, O2 saturation minute 6), gas exchange tests (alveolar-arterial oxygen gradient, blood gas analysis ), modification in dyspnea measured on the mMRC scale, the need for complementary oxygen therapy, etc… Radiological evolution (chest x-ray, HRCT) can also be useful (not essential, if not available comment as limitation). An example of a similar case where these concepts are addressed, which enrich the clinical case for the expert reader, has been recently presented by my research team (in case it serves as a guide) ^1^.

 It is also important to indicate whether adverse effects have appeared associated with any of the treatments.

 The CARE guidelines also consider the patient's perspective. Is it possible to ask the patient how she feels? In the case discussed by my research team ^1^, after adequate treatment with inhaled GM-CSF, a patient with aPAP told us “I take a deep breath and I notice that my chest fills with air to the bottom, not like before.”

 Without further ado, I congratulate you for the great work and for sharing it with the scientific community.

Are enough details provided of any physical examination and diagnostic tests, treatment given and outcomes?

Partly

Is the case presented with sufficient detail to be useful for other practitioners?

Yes

Is sufficient discussion included of the importance of the findings and their relevance to future understanding of disease processes, diagnosis or treatment?

Yes

Is the background of the case’s history and progression described in sufficient detail?

Yes

Reviewer Expertise:

clinical pharmacology

I confirm that I have read this submission and believe that I have an appropriate level of expertise to confirm that it is of an acceptable scientific standard, however I have significant reservations, as outlined above.

## References

[1] : Response to inhaled granulocyte-macrophage colony-stimulating factor in patient with mild-to-moderate autoimmune pulmonary alveolar proteinosis-24 months of follow-up. *Clin Respir J* .2023;17(10) : 10.1111/crj.13650 1077-1081 10.1111/crj.13650 37300395 PMC10542988

# Reviewer report: Reviewer response for version 2

**DOI:** 10.5256/f1000research.154600.r199233

**Published:** 2024-05-13

**Authors:** Valentin Coirier

**Affiliations:** 1 Rennes University Hospital, Rennes, France

I read with interest the case-report "the first reported case of pulmonary alveolar proteinosis with myasthenia gravis in a 27-year-old patient.  

 The text is well written, with a clear description of the case. We can find typical characteristics of the disease, without any doubt about the diagnosis. We can ask why the biopsy has been performed because chest scan was typical, as was liquid from BAL. But images are nice, and interesting to describe the case. 

 Authors have taken account of the comments from the first reviewing.

Are enough details provided of any physical examination and diagnostic tests, treatment given and outcomes?

Yes

Is the case presented with sufficient detail to be useful for other practitioners?

Yes

Is sufficient discussion included of the importance of the findings and their relevance to future understanding of disease processes, diagnosis or treatment?

Yes

Is the background of the case’s history and progression described in sufficient detail?

Yes

Reviewer Expertise:

interstitial lung diseases

I confirm that I have read this submission and believe that I have an appropriate level of expertise to confirm that it is of an acceptable scientific standard.

# Reviewer report: Reviewer response for version 2

**DOI:** 10.5256/f1000research.154600.r198480

**Published:** 2023-08-22

**Authors:** Helmi Ben Saad

**Affiliations:** 1 Faculty of Medicine of Sousse, Laboratory of Physiology, University of Sousse, Sousse, Tunisia

The authors have addressed all my remarks. Good job.

Are enough details provided of any physical examination and diagnostic tests, treatment given and outcomes?

Yes

Is the case presented with sufficient detail to be useful for other practitioners?

Yes

Is sufficient discussion included of the importance of the findings and their relevance to future understanding of disease processes, diagnosis or treatment?

Yes

Is the background of the case’s history and progression described in sufficient detail?

Yes

Reviewer Expertise:

Physiology and pulmonary function

I confirm that I have read this submission and believe that I have an appropriate level of expertise to confirm that it is of an acceptable scientific standard.

# Reviewer report: Reviewer response for version 1

**DOI:** 10.5256/f1000research.139794.r158819

**Published:** 2023-01-05

**Authors:** Helmi Ben Saad

**Affiliations:** 1 Faculty of Medicine of Sousse, Laboratory of Physiology, University of Sousse, Sousse, Tunisia

I read with a great interest the case report entitled “Case Report: The first reported case of pulmonary alveolar proteinosis with myasthenia gravis in a 27-year-old patient”.

 The case report is very interesting. However, some minor changes should be applied before the acceptance of the paper:

**POINT 1.** I strongly recommend applying the CARE guideline ^1^.

 For that reason, authors are asked to:

- Consult the guidelines (see PDF sent as an appendix)
- Submit (as an appendix) the CARE checklist (see the PDF)
- Write (in the beginning of the section Case report, page 3/7): This case reported was presented according to the CARE guideline (add the reference inside the paper and in the references list)

**POINT 2.** Abbreviations misuse: all abbreviations should be explained the first time they are used - unless it is a standard unit of measurement - and thereafter the use of abbreviations should be consistent throughout the paper.

**Example 1: Abstract**

 Please abbreviate Pulmonary alveolar proteinosis as PAP at first use (1 ^st^ line of the abstract) and therefore use always PAP.

**Example 2: Abstract and text**

 Define COVID-19 (and do not use the abbreviations COVID).

**POINT 3.** Key words

 Please avoid citing as keywords some terms previously used in the title or the abstract.

 Please opt for MeSH terms and classify the keywords in alphabetical order.

**POINT 4.** Case report section

 Line 17 page 3: In the sentence “The physical examination and the laboratory findings were unremarkable”: please name the laboratory tests that were performed.

 Line 27 page 3: can you add the numerical values (and normal range) of c-antineutrophil cytoplasmic antibodies and anti-Mi-2 antibodies?

 Last line of page 3: change the sentence “of 70% after a 6-min walk of 100 meters” by “of 70% after walking 100 meters during the 6-min walk test”.

**POINT 5.** Discussion

 The first sentence should be a reminder of the main result (to take home message from this case report)

 Page 5 line 7: why 2009 why not 2023? In other terms, how many cases were published in PubMed until 2023?

 Add some limitations of this case-report: for example “assessment under corticosteroids and azathioprine”, other limitations.

**POINT 6.** Consent (page 6)

 Change “Written informed consent for publication of their clinical details and clinical images was obtained from the patient” By “Written informed consent for publication of her clinical details and clinical images was obtained from the patient.”

**POINT 7.** References (page 6)

 Standardize the way you present the journal short name (sometimes use of point, sometimes no use of point).

Are enough details provided of any physical examination and diagnostic tests, treatment given and outcomes?

Yes

Is the case presented with sufficient detail to be useful for other practitioners?

Yes

Is sufficient discussion included of the importance of the findings and their relevance to future understanding of disease processes, diagnosis or treatment?

Yes

Is the background of the case’s history and progression described in sufficient detail?

Yes

Reviewer Expertise:

Physiology and pulmonary function

I confirm that I have read this submission and believe that I have an appropriate level of expertise to confirm that it is of an acceptable scientific standard, however I have significant reservations, as outlined above.

## References

[1] : The CARE Guidelines: Consensus-based Clinical Case Reporting Guideline Development. *Glob Adv Health Med* .2013;2(5) : 10.7453/gahmj.2013.008 38-43 10.7453/gahmj.2013.008 24416692 PMC3833570

# Response

**Published:** 2023-08-12

**Authors:** LENDA BEN HMIDA

**Affiliations:** Military hospital of Tunis Tunisia, Tunisia

I read, with a considerable attention, your review of the case report entitled “Case Report: The first reported case of pulmonary alveolar proteinosis with myasthenia gravis in a 27-year-old patient”.

 Thank you for revising our article and improving its quality. We addressed the comments mentioned in the review and we made some changes as stated. We ensured that the case report adheres to CARE guideline.

 We submitted accordingly our new version with great expectations.

 Here we respond to the changes that should be applied before the acceptance of the paper:

**POINT 1:**

 We applied carefully CARE guideline:

- We re-consulted the CARE guidelines of 2013.
- We submitted the CARE checklist (as an appendix).
- We Wrote (in the beginning of the section Case report, page 3/7): “This case reported was presented according to the CARE guideline”. We added the reference inside the paper in the references list.

**POINT 2:**

 We deeply regret our inattention concerning abbreviations misuse.

 Example 1: We defined our abbreviation PAP referring to Pulmonary alveolar proteinosis in the abstract.

 Example 2: We defined “COVID-19” by changing the term to “Coronavirus disease 2019” (without use the abbreviations).

**POINT 3:**

 We opted for MeSH terms and changed the keyword (used in the title or abstract) from:

- “Pulmonary alveolar proteinosis” to “Pulmonary alveolar lipoproteinosis”
- “Myasthenia gravis” to “myasthenia”
- “Rituximab” to “anti-CD20”

We classified the keywords in alphabetical order.

**POINT 4:**

Line 17 page 3: In the sentence “The physical examination and the laboratory findings were unremarkable”: We named the laboratory tests that were performed.

Line 27 page 3: We added the numerical value (and normal range) of c-antineutrophil cytoplasmic antibodies and anti-Mi-2 antibodies.

Last line of page 3: We changed the sentence “of 70% after a 6-min walk of 100 meters” by “of 70% after walking 100 meters during the 6-min walk test”.

**POINT 5:**

 We added a first sentence in the discussion part as a reminder of the main result (to take home message from this case report).

Page 5 line 7:  In PubMed, there was no systematic review of pulmonary alveolar proteinosis until 2023. There was no article or review specifying the number of cases published in PubMed until 2023.

 We added another limitation to our case report: The follow-up period was short (only 6 months). There was not enough hindsight to assess the *long* *-* *term effectiveness* of *rituximab* *.*

**POINT 6:**

 We changed “Written informed consent for publication of their clinical details and clinical images was obtained from the patient” by “Written informed consent for publication of her clinical details and clinical images was obtained from the patient.”

**POINT 7:**

 We standardized the way we present the journal short name (references list).
